# Acute Sickle Hepatic Crisis after Liver Transplantation in a Patient with Hb SC Disease

**DOI:** 10.1155/2015/761740

**Published:** 2015-02-19

**Authors:** J. H. Gillis, S. K. Satapathy, L. Parsa, P. B. Sylvestre, N. Dbouk

**Affiliations:** ^1^Department of Internal Medicine, The University of Tennessee Health Science Center, Memphis, TN 38104, USA; ^2^Department of Surgery, The University of Tennessee Health Science Center, Memphis, TN 38104, USA; ^3^Methodist University Hospital Transplant Institute, Memphis, TN 38104, USA

## Abstract

Acute sickle hepatic crisis (ASHC) has been observed in approximately 10% of patients with sickle cell disease. It occurs predominantly in patients with homozygous (Hb SS) sickle cell anemia and to a lesser degree in patients with Hb SC disease, sickle cell trait, and Hb S beta thalassemia. Patients commonly present with jaundice, right upper quadrant pain, nausea, low-grade fever, tender hepatomegaly, and mild to moderate elevations in serum AST, ALT, and bilirubin. We describe the case of a patient with a history of hemoglobin SC disease and cirrhosis caused by hepatitis C presenting approximately 1 year after liver transplantation with an ASHC. The diagnosis was confirmed by liver biopsy. Our patient was treated with RBC exchange transfusions, IV hydration, and analgesia and made a complete recovery. Only a limited number of patients with sickle cell disease have received liver transplants, and, to our knowledge, this is the first case of ASHC after transplantation in a patient with Hb SC disease.

## 1. Introduction

Acute sickle hepatic crisis (ASHC) is a clinical syndrome observed in sickle cell disease (SCD) that falls into a spectrum of hepatobiliary complications associated with sickle cell hepatopathy. The prevalence of ASHC is not well documented but may occur in up to 10% of patients with SCD [1]. As with all types of sickle cell hepatopathy, it occurs predominately in patients with homozygous SCD and to a lesser degree in patients with Hb SC disease, sickle cell trait, and Hb S beta thalassemia. Patients typically present with acute right upper quadrant pain, jaundice, low-grade fever, tender hepatomegaly, and nausea. Serum AST and ALT levels are usually less than 300 IU/L and total bilirubin rarely exceeds 15 mg/dL [1]. ASHC is usually self-limited, resolving with analgesia and intravenous fluid administration. In more severe cases, RBC exchange transfusions may be necessary. The incidence of ASHC in the transplanted liver is unknown, as very few patients with SCD have undergone liver transplantation [2].

## 2. Case Report

Our patient is a 56-year-old African American female with a history of sickle cell disease (HbSC) and chronic hepatitis C. She acquired hepatitis C from a remote blood transfusion which she received during a sickle crisis. She was treated with Peg IFN and ribavirin many years prior to transplant but unfortunately was a null responder. Her liver disease progressed to cirrhosis and was complicated by portal hypertension, refractory ascites, and recurrent episodes of SBP. The patient was also being followed by a hematologist locally since her diagnosis with SCD and required exchange transfusions several times a year for pain crises. She received a liver transplant at our institution with no major perioperative complications. Our patient did well on follow-up except for occasional pain crises which were mild and managed by the patient herself with over-the-counter analgesics. Her hemoglobin and hematocrit ranged between 7–9 and 22–29 g/dL, respectively, and she did not require any blood transfusions. Unfortunately she neglected to follow up with her hematologist during her first year after transplantation.

Approximately one year after transplantation, our patient presented with a 5-day history of jaundice, right upper quadrant pain, diffuse pruritis, and malaise. She denied any fever, chills, nausea, or bowel changes.

**Figure 1:**
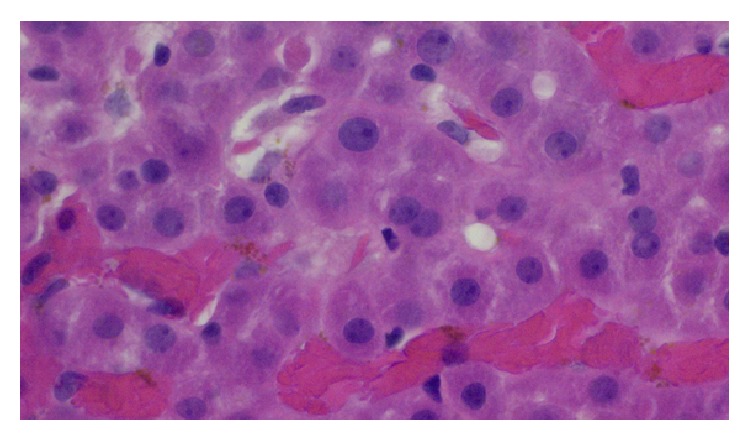
Liver biopsy (H&E stain), high power view. Note the presence of sickled red blood cells within the sinusoids and sinusoidal congestion by sickled red blood cells. There is also mild atrophy of hepatocytes, indicative of mild chronic hypoxia due to vascular flow impediment by aggregated sickled red blood cells.

**Figure 2:**
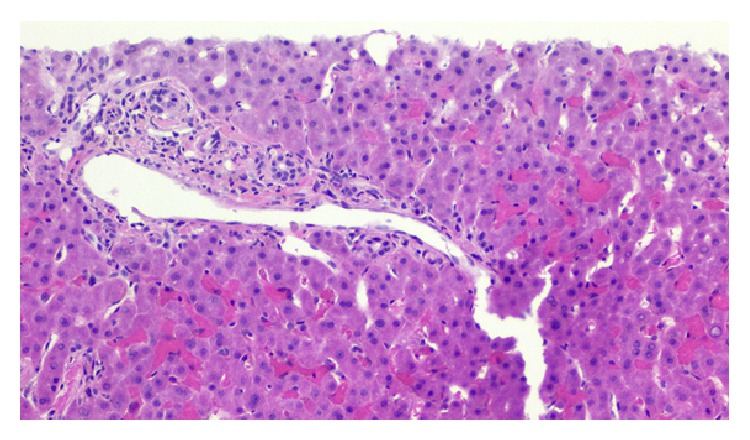
Liver biopsy (H&E stain), medium power view. The portal tracts are essentially histologically unremarkable. No histologic evidence of acute cellular rejection or recurrent hepatitis C infection is present.

Pertinent findings on physical exam included scleral icterus and exquisite right upper quadrant tenderness. Her initial laboratory findings were as follows: total bilirubin 3.3 mg/dL; direct bilirubin 2.5 mg/dL; AST 143 IU/L; ALT 159 IU/L; alkaline phosphatase 361 IU/L; GGT 416 IU/L; WBC 6,800; hemoglobin 9.9 g/dL; hematocrit 30%; platelets 346,000; and HCV RNA 254,000 IU/mL. Abdominal ultrasound with Doppler did not reveal any vascular abnormalities. A magnetic resonance cholangiography (MRCP) was suggestive of an anastomotic biliary stricture; however a subsequent ERCP did not reveal any abnormalities. A CT-guided needle biopsy of the liver was then performed and this revealed evidence of sinusoidal congestion and hepatocyte necrosis due to sickled RBCs as seen in Figure 1. In addition, there was no histologic evidence of recurrent hepatitis C or acute cellular rejection as seen in Figure 2.

Hematology was consulted to assist with management. Hemoglobin electrophoresis was requested and showed Hgb A 0%, Hgb A2 4.8%, Hgb F 1.4%, Hgb S 44.9%, and Hgb C 44.5%. Haptoglobin level was low (<8 mg/dL). Her absolute reticulocyte count and reticulocyte percent were both elevated at 102.6 thou/mcl and 3%, respectively. The patient underwent six cycles of RBC exchange transfusions. Hemoglobin electrophoresis profiles before and after RBC exchange transfusions are detailed in Table 1.

The patient's liver function tests improved significantly following exchange transfusions. Her symptoms also quickly improved over the following days and she was discharged home in stable condition with plans for follow-up with her hematologist for monthly RBC exchange transfusions. Labs on discharge were as follows: bilirubin: 1.6, ALT: 58, AST: 33, alkaline phosphatase: 121, Hgb: 10.6, and hematocrit: 31.

## 3. Discussion

Hepatic dysfunction caused by SCD is well known but difficult to define and thus incompletely documented. Experience with hepatic dysfunction in SCD patients who have undergone liver transplantation is even more limited as less than 25 cases of liver transplantation in patients with SCD have been documented [2]. Diagnosis is often challenging as patients typically have other contributing factors to liver dysfunction such as transfusion related iron overload, gallstone disease, and, in our patient, possible graft rejection and recurrent hepatitis C.

In our patient, diagnosis was confirmed by a liver biopsy and improvement in the patient's symptoms was ultimately realized with the use of RBC exchange transfusions. Although some have suggested that liver biopsies carry a high risk of complications in patients with SCD including an increased risk of bleeding and liver rupture [3], a liver biopsy remains an important and often essential diagnostic modality to evaluate abnormal LFTs particularly amongst transplant patients. In patients at a higher risk of complications, such as those with ascites, thrombocytopenia, or coagulopathy, a transjugular approach should be considered. It should be noted that published Guidelines from the American Association for the Study of Liver Disease do not list SCD or ASHC as a contraindication to performing percutaneous liver biopsies [4].

To our knowledge, this is the first case of ASHC reported in a patient with Hb SC disease. One case of ASHC after transplantation in a patient with sickle beta thalassemia has been described by Perini et al. [5]. As experience with liver transplantation in SCD patients continues to progress, the role of RBC exchange transfusions, both as a preventative measure and for management of acute conditions such as ASHC, should be further investigated. Risk stratification in patients who are homozygous for the disease versus those with Hb SC disease, Hb S beta thalassemia, and sickle cell trait may also prove to be of benefit.

## Figures and Tables

**Table 1:** 

Hemoglobin electrophoresis	Before RBC exchange transfusions (%)	After RBC exchange transfusions (%)
Hgb A	0	68.3
Hgb A2	4.8	2.7
Hgb F	1.4	<1
Hgb S	44.9	10.3
Hgb C	44.5	10.1
Hgb D and E	0	0
Hgb other^*^	4.4	8.6

^*^Bound hemoglobin A including glycosylated, carbamylated, or other bound hemoglobin types.
